# The Interplay between Gut Microbiota and Oral Medications and Its Impact on Advancing Precision Medicine

**DOI:** 10.3390/metabo13050674

**Published:** 2023-05-21

**Authors:** Sara Mousa, Muhammad Sarfraz, Walaa K. Mousa

**Affiliations:** 1 College of Pharmacy, Al Ain University, Abu Dhabi P.O. Box 112612, United Arab Emirates; 202010763@aau.ac.ae (S.M.); muhammad.sarfraz@aau.ac.ae (M.S.); 2 College of Pharmacy, Mansoura University, Mansoura 35516, Egypt

**Keywords:** drug metabolism, drug breakdown, microbiota, bioavailability, precision medicine

## Abstract

Trillions of diverse microbes reside in the gut and are deeply interwoven with the human physiological process, from food digestion, immune system maturation, and fighting invading pathogens, to drug metabolism. Microbial drug metabolism has a profound impact on drug absorption, bioavailability, stability, efficacy, and toxicity. However, our knowledge of specific gut microbial strains, and their genes that encode enzymes involved in the metabolism, is limited. The microbiome encodes over 3 million unique genes contributing to a huge enzymatic capacity, vastly expanding the traditional drug metabolic reactions that occur in the liver, manipulating their pharmacological effect, and, ultimately, leading to variation in drug response. For example, the microbial deactivation of anticancer drugs such as gemcitabine can lead to resistance to chemotherapeutics or the crucial role of microbes in modulating the efficacy of the anticancer drug, cyclophosphamide. On the other hand, recent findings show that many drugs can shape the composition, function, and gene expression of the gut microbial community, making it harder to predict the outcome of drug-microbiota interactions. In this review, we discuss the recent understanding of the multidirectional interaction between the host, oral medications, and gut microbiota, using traditional and machine-learning approaches. We analyze gaps, challenges, and future promises of personalized medicine that consider gut microbes as a crucial player in drug metabolism. This consideration will enable the development of personalized therapeutic regimes with an improved outcome, ultimately leading to precision medicine.

## 1. Introduction

**Figure 1:**
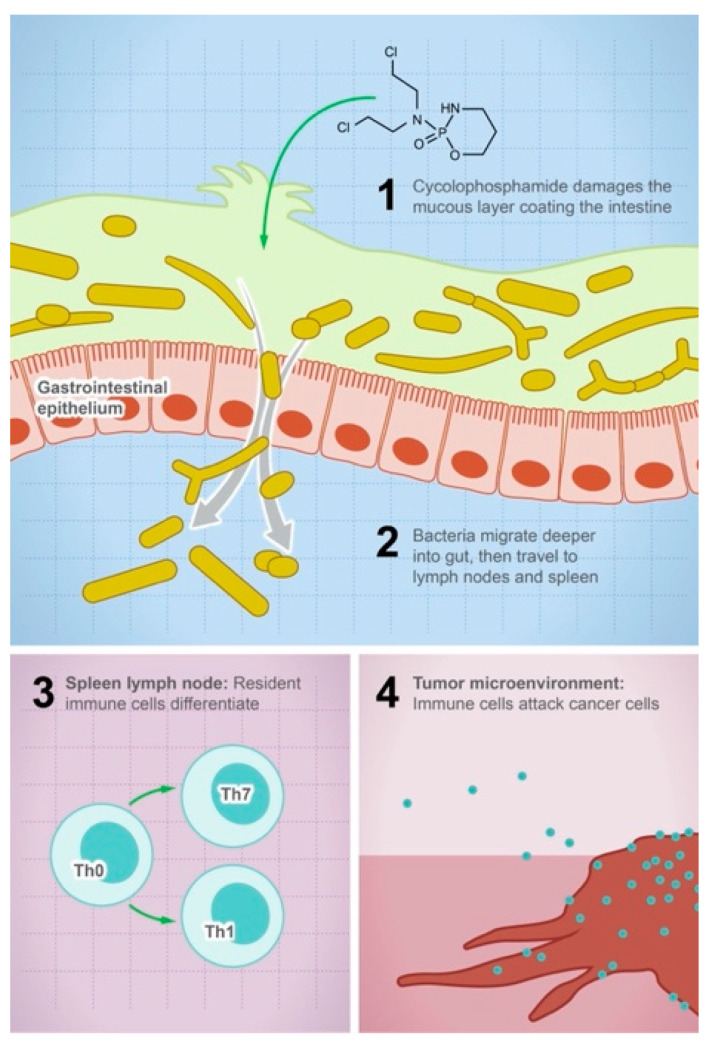
Gut microbes mediate the efficacy of the anticancer drug cyclophosphamide. The illustration shows the steps leading to the anticancer activity of cyclophosphamide: (1) the drug damages the mucous layer coating the intestine, (2) bacteria can systemically migrate to lymph nodes and spleen, (3) activation of the immune cell differentiation and production of cytokines and toxic mediators, and (4) the density of immune cells in the tumor microenvironment will increase and attack tumor cells.

Orally administered drugs encounter millions of microbial species in the gastrointestinal tract (GIT). With approximately 3.3 million unique genes, gut microbes are considered an invisible organ that vastly expands the human enzymatic capacity [1,2,3]. These microbes, their genetic components, and epigenetics regulation are unique to each individual and contribute to varied drug responses between individuals [3,4,5,6]. Several examples in the literature have noted the effect of microbes on drug metabolism [7,8,9]. Their effect is widespread over drugs used to treat cancer, depression, Parkinson’s disease, and cardiovascular diseases [10,11,12,13,14]. For example, *Mycoplasma hyorhinis* encodes an enzyme known as cytidine deaminase which deactivates the anticancer drug gemcitabine to 2′,2′difluorodeoxyuridine, contributing to chemotherapeutic resistance [15,16]. Other microbes such as *Escherichia coli* can metabolize multiple anticancer drugs such as gemcitabine, doxorubicin, and tretazicar [5,16,17]. A recent finding suggests that a single microbe, *Bacteroides thetaiotaomicron*, is capable of metabolizing more than 40 drugs including the widely used calcium channel blocker, diltiazem [7]. Computational software has been developed to predict the susceptibility of different drugs to microbial metabolism based on the presence of specific functional groups in the drug. Moreover, gut microbes might affect the efficacy of drugs in an indirect way, such as in the case of cyclophosphamide, where the activity of the drug is mediated by its damaging effect on the gut mucosa which enables the systemic translocation of gut microbes to lymph nodes and spleen, increasing the density of immune cells at the tumor microenvironment and activating an immune attack on cancer cells [18,19] (Figure 1). The interaction between gut microbes and oral medications is not a one-way interaction that makes these medications susceptible to microbial degradation, it is bidirectional, where drugs can also exert an effect on gut microbes shaping their composition and function [20]. It is generally acknowledged that gut microbes are affected by antibiotics, but more recent findings report that more than 24% of non-antibiotic drugs exert an antibiotic-like action on gut microbes [21]. For example, analysis of the gut microbial community in patients receiving metformin treatment revealed that metformin specifically decreases the abundance of *Bacteroides fragilis* [22], while atorvastatin promotes the growth of *Akkermansia muciniphila* and *Faecalibacterium prausnitzii*, and inhibits the growth of Proteobacteria and Enterobacteriacae. This interaction is also observed with supplements such as Vitamin D, which increases *Streptococcus salivarius*, *Bacteroides* sp., and Parabacteroides [23]. With increasing interest in pharmacomicrobiomics as an integral component of the personalized medicine approach [24,25], we present this overview of the current knowledge of microbial drug metabolism and its impact on drug bioavailability and therapeutic outcome. We discuss the bidirectional interactions between oral medications and gut microbes. We assess the recent development of computational tools to predict possible drug metabolism by microbes. Considering the unique microbiome signature of each individual in the planning of therapeutic regimes is a crucial component in the development of personalized and precision medicine [26,27].

## 2. Susceptibility of Oral Medications to Microbial Metabolism

**Figure 2:**
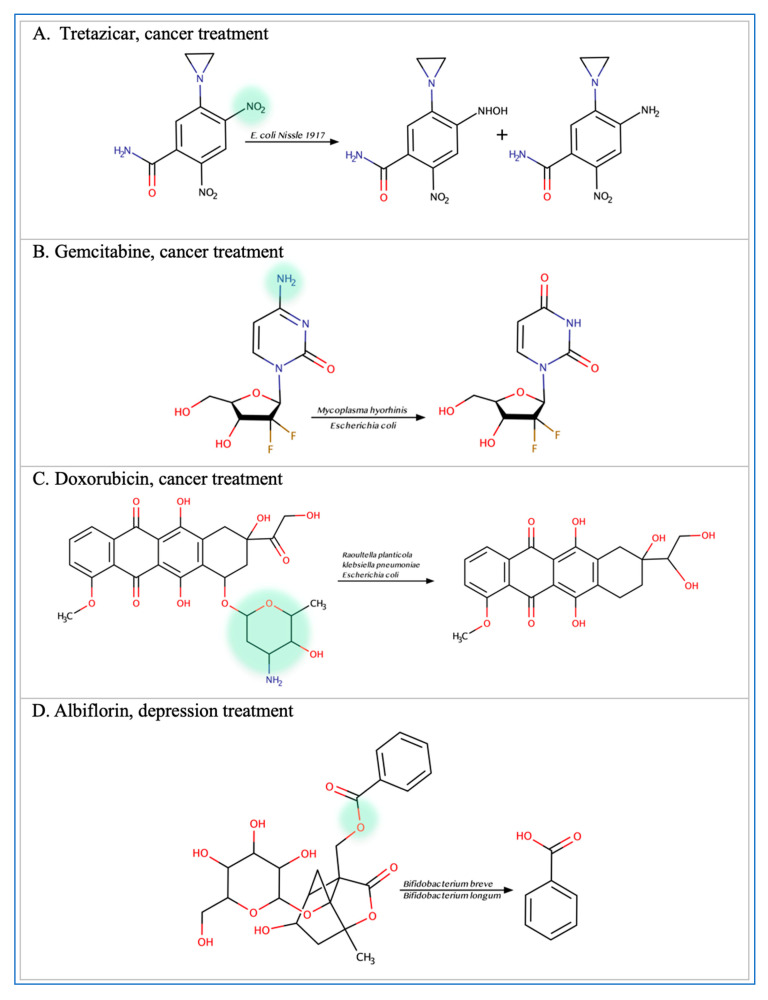

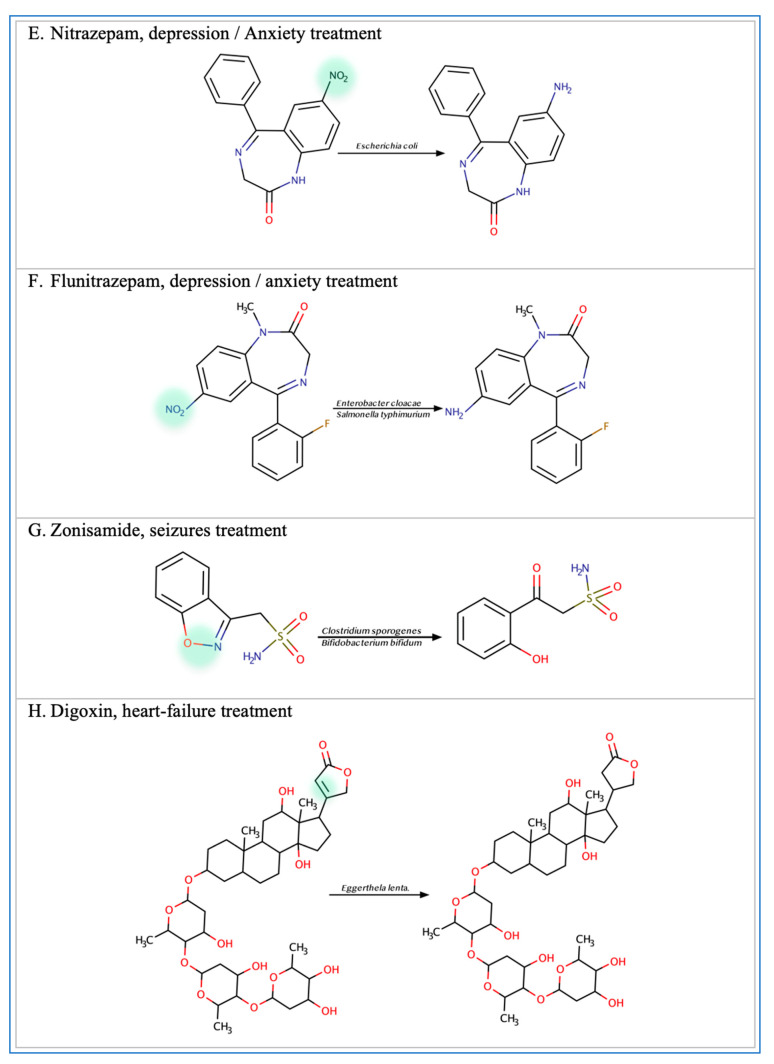

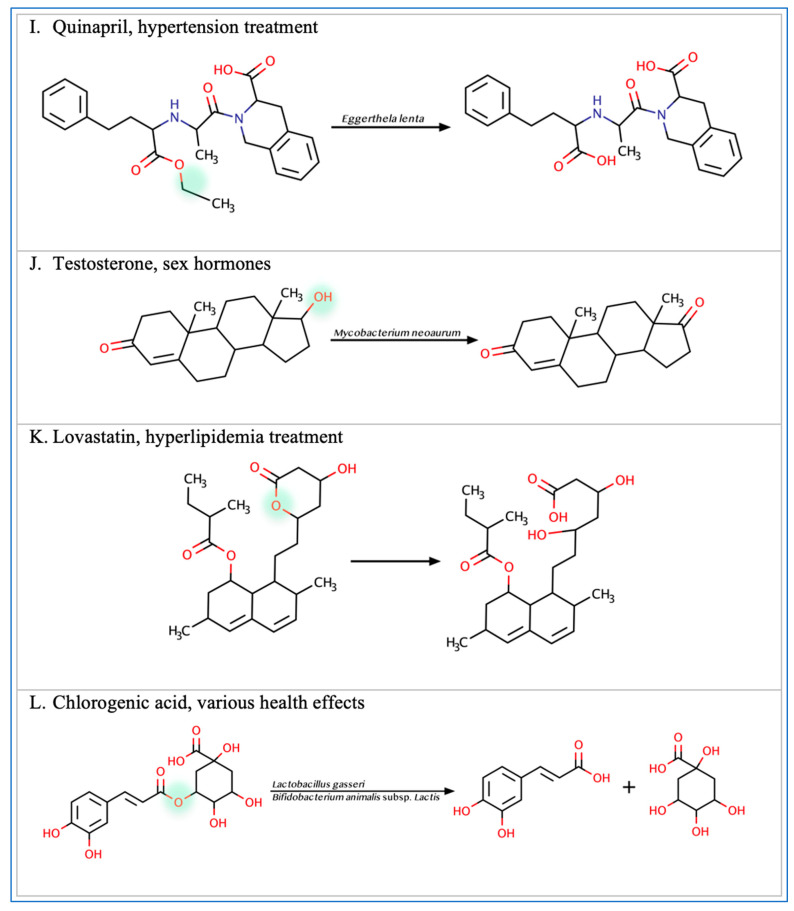
**Illustration of some examples of microbial drug metabolism.** (**A**) Shows degradation of the nitro functional group of tretazicar used for the treatment of cancer by *E. coli* Nissle 1917. (**B**) Shows degradation of the amine functional group of the anticancer drug gemcitabine by *Mycoplasma hyorihinis* and *E. coli*. (**C**) Shows deglycosylation of doxorubicin used for the treatment of cancer by *Raoultella planticola*, *Klebsiella pneumoniae*, and *E. coli.* (**D**) Shows degradation of the ester functional group of albiflorin used for the treatment of depression by *Bifidobacterium breve* and *Bifidobacterium longum.* (**E**) Shows degradation of the nitro functional group of nitrazepam used for the treatment of depression and anxiety by *E.coli.* (**F**) Shows degradation of the nitro functional group of flunitrazepam used for the treatment of depression and anxiety by *Enterobacter cloacae* and *Salmonella* typhimurium. (**G**) Shows degradation of the benzisoxazole N-O bond of zonisamide used for the treatment of seizures by *Clostridium sporogenes* and *Bifidobacterium bifidum.* (**H**) Shows a reduction in the carbon 20 and carbon 22 bonds of digoxin used for the treatment of heart failure by *Eggerthela lenta. (***I**) Shows degradation of the ester bond of quinapril used for the treatment of hypertension by *Eggerthela lenta.* (**J**) Shows dehydrogenation of the hydroxyl functional group at carbon 17 of testosterone by *Eggerthela lenta.* (**K**) Shows degradation of the lactone group of lovastatin used for the treatment of hyperlipidemia by a group of gut microbes. (**L**) Shows degradation of the ester functional group of chlorogenic acid by *Lactobacillus gasseri* and *Bifidobacterium animalis* subsp *lactis*.

Multiple functional groups within oral medications are susceptible to microbial metabolism leading to drug activation, inactivation, or toxicity. Recent reports show that particular functional groups such as ester, amide, nitro, and azo, cause the drug to be more susceptible to microbial enzymatic degradation [7]. For example, albiflorin contains an ester group which causes it to be susceptible to hydrolysis by some species of *Bifidobacteria* [13]. Similarly, benzodiazepines are susceptible to metabolism due to the presence of a nitro group [28,29]. The antibacterial activities of prontosil, neoprontosil, sulfasalazine, balsalazide, and olsalazine are mediated by microbial azo reduction [30,31,32,33,34]. The antidiarrheal effect of Loperamide oxide is mediated by cleavages of N-oxide bonds, and the production of Loperamide by intestinal microbiota [35]. The laxative effect of sodium picosulfate requires the conversion of bisulfate to 4,4′-dihydroxydiphenyl-(2-pyridyl)-methane by bacteria residing in the gut [36]. The diarrheal side effect of the anticancer drug Irinotecan is attenuated by the glucuronidase enzyme produced by intestinal flora [37]. Anaerobic incubation of levamisole with human gut microbes results in the production of levametabol-I, II and III, thiazole ring-opened metabolites that have been implicated in anti-colon cancer activity [38]. Selected examples of common microbial drug metabolism based on particular functional groups are listed in Table 1 and/or illustrated in Figure 2.

## 3. Microbial Drug Metabolism for Different Oral Medications Classes

Here we discuss examples of microbial metabolism based on xenobiotic intended use, including cancer, central nervous system, cardiovascular, steroids, supplements, and natural products.

### 3.1. Anticancer Drugs

**Figure 3:**
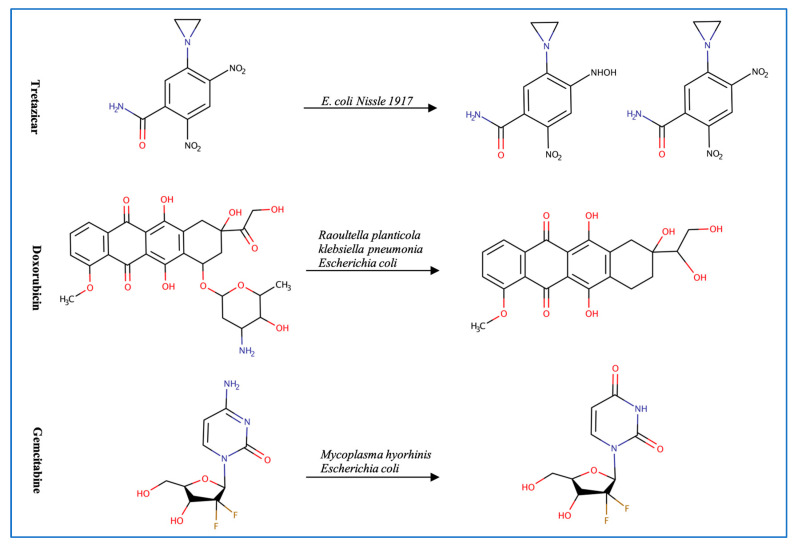
Examples of anticancer drugs transformed by gut microbes.

Inconsistent response and resistance to anticancer therapeutics is a major concern [4]. This resistance, in part, might be attributed to the microbiome microenvironment (Figure 3). Multiple microbial species have been linked to resistance to chemotherapeutics including *E. coli* and *Listeria welshimeri* [5]. *E. coli* is involved in the degradation of multiple anticancer drugs such as tretazicar, gemcitabine, and doxorubicin [5,16,17]. The strain *E. coli Nissle 1917* reduces tretazicar to its active metabolite [5]. *E. coli* produces cytidine deaminase enzyme which converts gemcitabine to the inactive product 2′,2′difluorodeoxyuridine [16,17]. The bacterial cytidine deaminase is also found in *M. hyorhinis* which metabolizes gemcitabine to 2′,2′-difluoro-2′-deoxyuridine, reducing the drug’s activity by 10-60-fold. The presence of pyrimidine nucleoside phosphorylase further potentiates the metabolism of gemcitabine, because it catabolizes the products from gemcitabine metabolism, increasing the reaction rate towards gemcitabine degradation [15]. Similarly, *Raoultella planticola* can convert doxorubicin to 7-deoxydixirubicinlone and 7-deoxydoxorubicinol by reductive deglycosylation [17]. Of note is that a molybdenum cofactor is required to degrade doxorubicin by multiple microbes such as *Raoultella planticola*, *K. pneumoniae*, and *E. coli* BW25113. In addition, gut microbes might have an indirect effect in enhancing response to anticancer drugs. Data show that the administration of antibiotics with the immune checkpoint inhibitor (ICI) anti-PD-1, decreases the progression-free survival (PFS) and overall survival (OS) period [49] in patients, while administration of *Alistipes indistinctus*, *Akkermansia muciniphila*, and *Enterococcus hirae*, restores efficacy of the treatment [50]. These microbes are thought to activate an immune response against cancer cells by increasing the secretion of IL-2 from the dendritic cells and recruiting different types of T cells in the tumor microenvironment [50,51]. The gut microbial signature can also be used as a biomarker indicative of possible ICIs’ treatment response. For example, a high fecal microbial diversity and abundance of the bacterial species *Enterococcus faecium*, *Veillonella parvula*, *Bifidobacterium longum*, *Bifidobacterium adolescentis*, *Lactobacillus* sp., *K. pneumoniae*, *Collinsella aerofaciens*, and *Parabacteroides merdae* suggest a better response [52]. Additionally, a longer progression-free survival period is linked to a high diversity and abundance of Romunicoccaceae, Clostridales, and *Faecalibacterium* [53]. The baseline microbiome can predict poor ipilimumab treatment response in metastatic melanoma patients with an accuracy of 99%. For example, the bacterial OTUs (*Bacteroides uniformis*, *Bacteroides vulgatus*, *Parabacteroides distasonis*) can act as potential biomarkers for ipilimumab-colitis free patients [54]. While previous findings support the effect of gut microbes on ICIs, they also highlight their potential role as biomarkers for treatment response. Given the crucial impact of these metabolic reactions on chemotherapeutics resistance, it is essential to consider the microbiome inter-patient variation and potential metabolic activities that affect treatment outcome [17].

### 3.2. Central Nervous System Drugs

**Figure 4:**
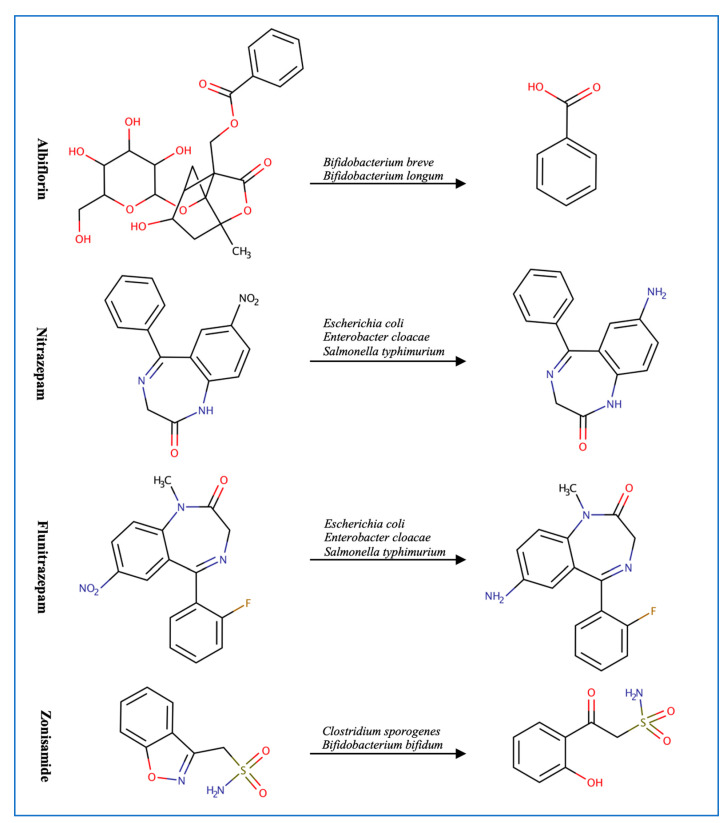
Examples of CNS drugs transformed by gut microbes.

Mounting evidence supports the link between gut microbes and neurodegenerative and psychological disorders [55,56,57] (Figure 4). For example, the bacterial strains *Bifidobacterium breve*, *Bifidobacterium longum*, *Bifidobacterium animalis*, and *Bifidobacterium adolescentis* can metabolize the anti-depression drug, albiflorin. *B. longum* has the strongest ability at hydrolyzing albiflorin followed by *B. breve* since they are the only two microbes that significantly increase the concentration of albiflorin’s metabolite. After searching the genome of the strains for esterase enzymes and evaluating the 3D models of the proteins, similarities were found in the core domain, indicating that esterase enzymes could be responsible for albiflorin conversion [13]. It is important to highlight the common use of those hydrolyzing bacterial strains in probiotic supplements since it could interfere with treatment response. The metabolizing effect is not limited to *Bifidobacteria* since gut microbes can metabolize clonazepam, nitrazepam, and flunitrazepam through nitro-reduction [28,29,58]. The genes *nfsB*, *nfnB*, and *nfsI* are responsible for the metabolic effect of *E. coli*, *Enterobacter cloacae*, and *Salmonella* typhimurium, respectively [29]. Reports show that *Clostridium sporogenes* and *Bifidobacterium bifidum* can also reduce zonisamide and risperidone through the benzisoxazole N-O bond into inactive forms [59].

**Figure 5:**
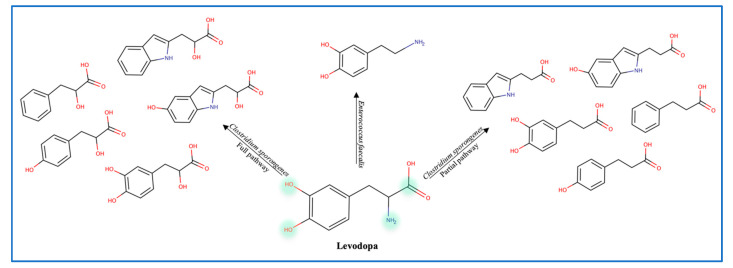
Levodopa metabolites produced by gut microbes.

To explain the variations in levodopa (L-dopa) treatment response, the role of gut microbes was evaluated by administering antibiotics to patients receiving the treatment. Data show that antibiotics increase the efficacy of levodopa, which confirms the role of gut microbes in metabolizing L-dopa [29]. Balskus et al. identified a decarboxylase enzyme in *Enterococcus faecalis* as the main metabolizing enzyme. However, this decarboxylation is non-selective since both L-dopa and tyrosine are decarboxylated (Figure 5) [12]. Moreover, the tyrDc operon is deemed essential for the *E. faecalis* decarboxylating effect [60]. Further conversion of dopamine to m-tyramine is carried out by another gut microbe known as *Eggerthela lenta* which produces a dopamine dehydroxylase molybdenum-dependent enzyme. Interestingly, co-administration of carbidopa (a tyrosine decarboxylase inhibitor) was found to be partially effective against bacterial tyrosine decarboxylase (inhibiting 50% of *E. faecalis* enzyme) [12]. L-dopa is also deaminated by *Clostridium sporongenes* to propionic acid metabolites through the activity of aromatic amino transferase. Silencing of this enzyme resulted in partial deamination and reduction in propionic acid metabolites. Although even with partial deamination these metabolites can still inhibit ileal muscles motility [61,62]. Of note is that Levodopa metabolites produced by *C. sporongenes* are either fully deaminated or partially deaminated due to a mutated dehydratase enzyme in the deamination pathway. These findings emphasize the need to consider the microbiome variation between individuals and adjust the dose of levodopa accordingly.

### 3.3. Cardiovascular Drugs

**Figure 6:**
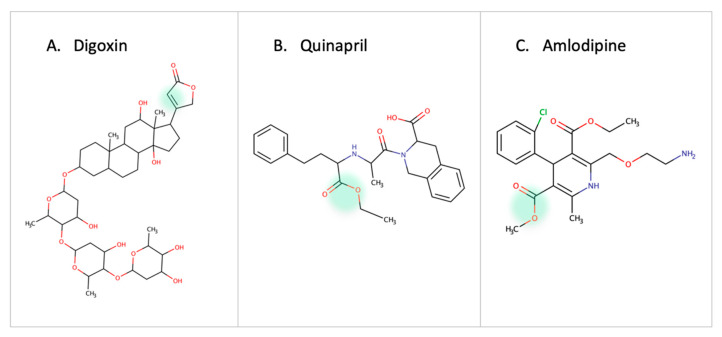
Examples of cardiovascular drugs transformed by gut microbes.

One of the earliest reports on drug-microbe metabolism was on Digoxin. It is reduced in the gut by *Eggerthela lenta* to dihydroxy digoxin through a mechanism similar to that of the natural substrate fumarate by a cardiac glycoside reductase enzyme [63,64,65]. In hypertension patients, the use of quinapril (ACEI, angiotensin converting enzyme inhibitor) or amlodipine (CCB, calcium channel blocker) is affected by esterase enzymes in gut microbes [10,66] (Figure 6). Administration of antibiotics in patients receiving Amlodipine led to an increase in the drug concentration which proved the role of microbes in amlodipine degradation. Accordingly, it is necessary to adjust the dose of quinapril and amlodipine in patients receiving antibiotics or with high/low abundance of the involved bacterial strain [66]. The treatment response of lovastatin is altered due to the presence of gut microbes. Administering the drug with antibiotics decreases the active hydroxy acid metabolite. This highlights the role of gut microbes in metabolizing statins and the indirect drug–drug interaction between statins and antibiotics [11].

### 3.4. Steroids and Corticosteroids

**Figure 7:**
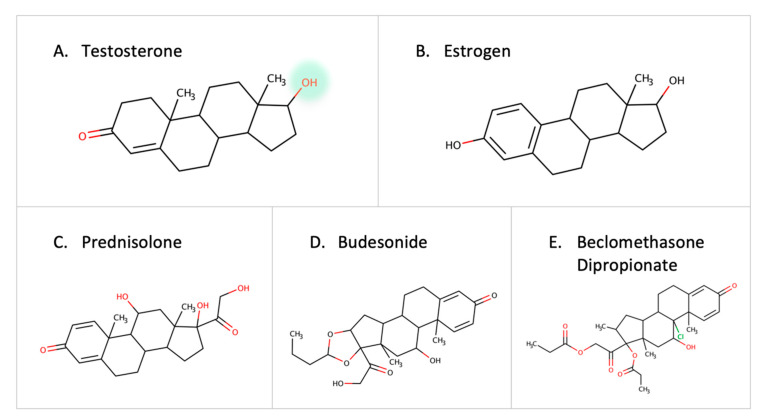
Examples of steroids and corticosteroids transformed by gut microbes.

Steroid-degrading enzymes are present in *Proteobacteria* and *Actinobacteria* [67]. Recently, the testosterone-degrading enzyme, 3-beta-hydroxysteroid dehydrogenase enzyme (3beta-HSD), was identified in *Mycobacterium neoaurum* and other microbes [68]. A low level of the natural testosterone can result in depression-like symptoms in males [68,69,70]. Thus, the abundance of microbes containing steroid-degrading enzymes can be a contributor to disease progression. Other steroid-degrading enzymes such as steroid reductase and hydroxysteroid dehydrogenase are expressed by *Streptococcus mutans* and *Bacillus cereus* [71]. Moreover, the desmolase enzyme in *Clostridium scindens* is capable of cleaving the side chain of 17-hydroxylatedcorticoids [72]. Estrogen is inactivated by glucuronidation, but gut microbes reactivate it by eliminating the glucuronic moiety due to the effect of B-glucuronidase [73]. Since estrogen is implicated in breast cancer, it is necessary to further investigate the role of gut microbes in estrogen metabolism [74]. In addition to native hormones, reports show that colonic bacteria can metabolize external corticosteroids such as prednisolone, budesonide, and beclomethasone dipropionate before they reach the site of action [75] (Figure 7). With the established links between steroid hormones and certain diseases [76], gut microbes can play a major role in degrading drugs, leading to a decrease in drug response or contributing to the pathogenesis of certain diseases.

### 3.5. Miscellaneous Xenobiotics and Natural Substances

Gut microbes can exert an effect on supplements, such as vitamin D. Gut microbes have been linked to low vitamin D levels since germ-free mice have shown high levels of fibroblast growth factor 23 (FGF23), while microbial transplantation restored vitamin D and FGF23 balance, which might suggest an indirect effect of gut microbes [77] although results are conflicting [78]. Additionally, gut microbes also affect natural substances such as chlorogenic, gallic, ferulic, and caffeic acids. Chlorogenic acid has various effects such as antioxidant [79], anti-inflammatory [79], anti-hypertensive [80], and glucose regulation [81]. An ongoing debate is whether chlorogenic acid can inhibit the growth of certain microbes or if the microbes are the ones capable of degrading the compound using enzymes such as cinnamoyl esterase. Faulds et al. reported that *Lactobacillus gasseri* can use phenolic compounds as its carbon source, which further stimulates its microbial growth. Upon testing microbes’ ability to degrade phenolic compounds, *L. gasseri* and *Bifidobacterium animalis* subsp. *Lactis* have been identified as the microbes with the greatest chlorogenic acid degrading effect. The mentioned strains contain cinnamoyl esterase that degrades chlorogenic acid to caffeic acid and quinic acid [82]. Munoz et al. reported that *Lactobacillus brevis* isolated from human feces CECT 4121, human mouth CECT 5354, and wine fermentation RM84, can decarboxylate p-coumaric acid, ferulic acid, caffeic acid, gallic acid, and protocatechuic acid, to vinyl phenol, vinyl guaiaol, vinyl catechol, pyrogallol, and catechol, respectively, but subsequent modification of the vinyl derivatives did not occur. Although p-coumaric acid and caffeic acid were completely decarboxylated, ferulic acid was still present, indicating non-complete decarboxylation. After searching the genome of *L. brevis* CECT 5354, a sequence encoding a phenolic acid decarboxylase explained the observed effect [83].

## 4. Effect of Oral Medications on Gut Microbes

**Figure 8:**
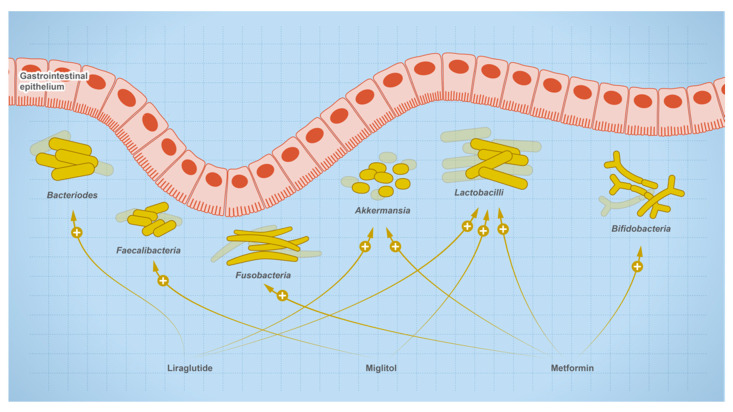
Effect of therapeutics used for the treatment of type-2 diabetic on gut microbial abundance.

The relationship between gut microbes and drugs is bidirectional, where drugs are susceptible to microbial metabolism and can shape the composition of the microbial community, such as drugs used for the treatment of type 2 diabetes (Figure 8). Users of metformin showed elevated levels of butanoate, quinone, and sugar breakdown metabolites with species of Enterobacteriaceae such as *E. coli* being the main contributors to these functional shifts in metformin users. Other research shows that mice on a high-fat diet (HFD) experienced dysbiosis when treated with metformin, while untreated HFD mice showed an increase in the genera *Akkermansia* and *Alistipes*, and a decrease in *Lactonifactor*, *Lactococcus*, *Anaerotrotruncus*, *Parabacteroides*, *Odoribacter*, *Lawsonia*, and *Blautia.* In addition, one of the experienced metformin treatment outcomes can be due to the role of *Akkermansia* as an anti-inflammatory. It reduces the expression of IL-6, IL-1b, mRNA, and normalizes the levels of regulatory T cells [84]. In newly treated T2D patients, microbial analysis of fecal samples collected after 3 days of metformin treatment showed a reduction in the *Bacteroides* genus. Other research showed metformin also reduces the growth of *B. fragilis* and this effect is mediated through the folate and methionine pathway [22]. Metformin increases the levels of 5-methyl tetrahydrofolate, 5,10-methylene tetrahydrofolate, S-adenosylmethionine, and S-adenosylhomocysteine, but reduces the levels of methionine and tetrahydrofolate [85]. The observed effect was consistent with methionine synthase inhibition in which 5-methyl tetrahydrofolate is accumulated [86]. Accordingly, metformin may cause its effect through a similar inhibition.

Interestingly, in a meta-analysis study, it was found that changes in the abundance of specific individual microbial taxa were associated with multiple independent drugs, while individual microbial features were also associated with single drugs [87,88]. For example, *Streptococcus salivarius* abundance increased in opiates, oral steroids, platelet aggregation inhibitors, proton pump inhibitors (PPIs), SSRI antidepressants, and vitamin D supplements [88], while benzodiazepine was found to be associated with an increase in the abundance of *Haemophilus parainfluenzae*, a bacterium that has been reported to be more common in patients with irritable bowel syndrome [87]. Increased abundances of *Bifidobacterium dentium* were found selectively in PPI users, while *Eubacterium ramulus* was in abundance within participants on SSRI antidepressants, and tricyclic antidepressant users showed higher abundances of *Clostridium leptum*. Individuals taking laxatives showed higher abundances of *Alistipes* and *Bacteroides* species [87] and steroid inhaler users had *Streptococcus mutans* and *Bifidobacterium dentium* in greater abundance. The use of drugs was also associated with shifts in gut function profiles [88]. For example, individuals taking high doses of PPIs demonstrated a significant decrease in a pathway involved in amino acid biosynthesis. In a group of patients treated with atorvastatin, the drug decreased the relative abundance of the following taxa and genera: Proteobacteria, Enterobacteriaceae, Desulfovibrio, *Prevotella*, *Collinsella*, and *Streptococcus*, while it increased the abundance of Firmicutes, *Akkermansia muciniphila*, and *Faecalibacterium prausnitzii* [20]. Moreover, the administration of methotrexate resulted in various alterations in the gut microbial community, especially in Bacteroidetes. Methotrexate consistently decreased *Bacteroidetes* in bacterial isolate, mice, and the gut community [89]. Analysis of fecal samples of subclinical thyroidism patients receiving L-thyroxine indicated the presence of a dose-dependent relationship between L-thyroxine and gut microbes. The drug changed the relative abundance of microbial species that have a role in hydrolysis and carbohydrate metabolism functions performed in the gut. Increasing the dose of L-thyroxine increased the genera *odoribacter*, *Enterococcus*, and *Ruminococcus*. Since the Enterococcus species is an antibiotic-resistant strain, it may increase the chance of patients developing infections. It is noteworthy to mention that *Ruminococcus* increased in patients with Hashimoto’s type of hypothyroidism [90].

A meta-analysis of cohort studies revealed that several drugs affect the gut microbial composition of patients. There was an association between certain drugs and the abundance of specific microbial species. The use of SSRIs, oral steroids, platelet aggregation inhibitors, opiates, and vitamin D increased the abundance of *Streptococcus salivarus*. The increase in *Eubacterium ramulus* was specifically associated with the use of SSRIs. This category of medication consisted of six drugs with paroxetine comprising 32% of it. Additionally, the use of laxatives was associated with an increase in the abundance of *Alistipes* and *Bacteroides*. Moreover, using Tricyclic antidepressants increased the abundance of *Clostridium leptum* and *Actinomyces*. The use of steroid inhalers was associated with an increase in the abundance of *Streptococcus mutans* and *Bifidobacterium dentium*. Unlike inhaled steroids, oral steroids increased the abundance of *Methanobrevibacter smithii* and the methanogenesis pathways. To explain the weight gain associated with oral steroids administration, researchers found an association between *Methanobrevibacter smithii*, obesity, and high BMI. One drawback to the study was the fact that some changes in the microbiome profile could be due to the disease itself, not just the drug [88]. Moreover, researchers reported an increase in the relative abundance of *Bacteroides* spp. and *Parabacteroides* spp. upon vitamin D supplementation, and a decrease in the *Firmicutes/Bacteroides* ratio. However, for the results to be conclusive, assessment of a larger sample size would be necessary to confirm the association [23]. Table 2 summarizes the effects of several drugs on gut microbes. Table 2 summarizes selected examples of drugs and their effect on the microbiota composition.

## 5. Computational Prediction of Microbial Drug Metabolism

There are some available tools that can predict possible drug metabolism but they face several obstacles since the microbiome is specific to each individual and changes with multiple factors such as diseases, drugs, and lifestyle [92]. Zeng et al. (2019) created a database to include information on the transformation of bioactive substances by gut microbes along with disease and microbe interactions. Only experimentally determined interactions are referenced in the database. Using the NCBI taxonomy database, the authors collected information on the identified microbial strains, drugs, bioactive substances, herbal medicines, traditional medicines, and environmental pollutants. Finally, they developed a prediction web server called “MASI: Microbiota Active-Substance Interaction database”. Based on the input in the search engine, related data from the literature are listed. For instance, if a drug name is searched, all microbes that metabolize this drug are listed [93]. MASI was followed by the database “MagMD: Metabolic Action of gut Microbiota to Drugs” which includes information on enzymes and their impact on drug efficacy. The database was developed in a process similar to MASI [94].

To predict the possibility of drug degradation by gut microbes, Zhao et al. (2017) developed a machine-learning prediction tool called “Drug Bug”. They analyzed 491 gut bacterial genomes for metabolic enzymes, leading to a total of 324,697 metabolic enzymes. The enzymes were distributed into specific metabolic enzyme (EC) classes that perform certain metabolic modifications. The substrates of the database metabolic enzymes were used to predict the drug molecules that may be degraded. After modifications of the prediction tool, a web server called “DrugBug” was developed, where three steps are performed to predict a molecule’s bacterial degradation. In the first step, the molecule’s PubChem ID is entered, or the mol/sdf file uploaded, then the model, sampling, and probability threshold are determined to predict the major EC class (Oxidoreductase EC1, Transferases EC2, Hydrolases EC3, Lyases EC4, Isomerases EC5, Ligases EC6). In the second step, the molecule’s EC-subclass is determined. In the third step, the fingerprint, Tanimoto index, and best protein hits parameters are selected. Lastly, microbes that have metabolic enzymes capable of degrading the molecule are listed [95]. Additionally, researchers evaluated the literature for drug-microbe interactions and collected around 455 confirmed interactions. Then they used machine learning to develop 11 models utilizing different techniques to predict drug and microbe interactions. The model sorts drugs into either depleted, or not depleted [96]. Another machine learning model, that includes 40 bacterial strains from the gut, trained over 18,600 drug/microbiome interactions that could predict the effect of administered drugs on the microbiome. Such a model is essential since variations in microbiome diversity are related to certain diseases [97]. The mentioned prediction software and databases are summarized in Table 3. Further development of machine learning tools to accurately predict possible microbial drug metabolism is crucial to advance the field of pharmacomicrobiomics.

## 6. Discussion

The gut microbial community encodes a plethora of enzymes that metabolize most drugs with various chemical structures, functional groups, and intended use. Moreover, the microbial community is also manipulated by oral medications that can promote or inhibit the growth of certain microbial strains, resulting in an indirect or unknown effect on body functions. Multiple databases and software have been developed to predict this complex relationship toward advancing personalized and precision medicine. The establishment of a systematically curated approach to defining the therapeutic outcome of each prescription drug based on the patient-microbiome signature will be the next frontier in advancing therapeutics’ efficacy and outcome. Furthermore, it is crucial to include the evaluation of drug–microbe interactions in the Food and Drug Administration (FDA) drug approval. This can be implemented through the evaluation of drug degradation by gut colonizing microbes, such as by using a fecal microbial extract, before the drug is approved for clinical trials [98].

### 6.1. The Implementation of Pharmacomicrobiomic in Therapeutic Regimes

Individuals differ widely in their genes that metabolize drugs and genes that encode for cellular receptors, resulting in varied pharmacokinetics and pharmacodynamics of prescribed drugs. The implementation of pharmacogenomics in drug prescription, although sporadic and not comprehensive for all patients and all drugs, shows promise in advancing healthcare. There is now another major player that has never been accounted for, namely, the microbiome. The development of personalized prescriptions based on an individual’s microbiome signature to predict response and resistance to specific drugs is referred to as pharmacomicrobiomics [24,25], which is a challenging yet promising advance in precision medicine. The availability of genomic and proteomic data will have a profound impact on accelerating the discovery of machine learning software to predict if a particular gene or a signature metabolite could be linked to a specific reaction leading to drug metabolism. Although multiple algorithms have been developed, they still need further improvement to address concerns such as accuracy and validity for translational medicine [99,100,101,102].

#### 6.1.1. Challenges of Microbiome-Based Personalized Medicine

The microbiome is very dynamic, even within the same individual, and is shaped by multiple factors including diet, lifestyle, diseases, drugs, stress, and host genetics [103,104]. This dynamic makes the drug–microbiota interaction harder to predict or evaluate based on species variability. Prediction of drug–microbiota interaction should be based on an encoded gene, not species, because bacterial genes are redundant among human microbiomes. Here, for example, we screened the presence of cytidine deaminase, originally identified in *Mycoplasma* sp., in other microbes. We identified 78 distinct species that harbor the same gene and, thus, can result in the same effect on gemcitabine or other drugs that share the same functional groups. Additionally, genes could be transferred from one species to another, especially under stress conditions, and the chronic use of drugs might be considered a stress-like condition for microbes [105]. Moreover, bacterial gene expression could also be induced in response to chronic drug administration. For example, research shows that microbial metabolism for the calcium channel blocker, diltiazem, is enhanced with repeated administration [7]. To add to the challenge, we must consider the possibility of cooperation between different microbes which leads to drug metabolism. For example, incubation of the steroid drug dexamethasone with 28 fecal cultures obtained from healthy human donors resulted in different metabolic products that were not dependent on culture density or the presence of the main dexamethasone metabolizing bacteria, *Clostridium scindens* [7]. Resources such as the second version of the assembly of gut organisms through reconstruction and analysis (AGORA-2) are very useful if utilized to create patient-personalized microbiome models. This will aid in predicting the possible degradation of administered drugs prior to starting the treatment plan [106].

#### 6.1.2. Promises of Considering the Microbiome Signature in Prescriptions

A simple modification to the prescription regime can result in a huge impact, for example, the use of enzyme inhibitors to suppress an undesirable microbial metabolism and thus decrease drug resistance. Further examples are the use of cytidine deaminase inhibitors in conjunction with gemcitabine to decrease resistance to chemotherapy [107] or the use of protease inhibitors with peptide drugs [108]. Another intervention might include the use of antibiotics to eliminate metabolizing microbes, for example, *Helicobacter* pylori decarboxylates levodopa in the gut before reaching the central nervous system, thus decreasing brain exposure and drug efficacy [109]. Studies showed that the elimination of *H.* pylori improved the absorption and pharmacokinetic properties of Levodopa [110]. On the other hand, to enable a desirable microbial metabolism, we can use probiotics, fecal transplants, or purified enzymes [111]. Another interesting approach is the manipulation of the microbiome through diet modification or the implementation of prebiotics to indirectly affect drug response, especially for chronically used drugs [112]. It is interesting to speculate that drug metabolism could also be affected by quorum sensing, the process that mediates microbial communication through secretion of small diffusible molecules known as autoinducers. Quorum sensing is affected by population density and might show an impact on drug metabolism either by its direct effect on some microbial cellular processes or indirectly thorough controlling expression of genes that encodes enzymes catalyzing drug breakdown. Manipulation of quorum sensing in microbes is now seen as a promising approach to control virulence, and it will also be interesting to investigate in the future how this process might precisely affect microbe–drug interactions [113,114,115,116]. To enable these interventions, the scientific community must advance the development of multi-omics-based machine learning models to accurately predict microbiota–xenobiotic interactions and further enable translational precision medicine [117].

## 7. Conclusions

Given the wide variation in microbiome signature between individuals, and their profound impact on metabolism of medications, implementation of pharmacomicrobiomics in medicine should be considered. Moreover, early pharmacokinetics studies should be revised to include microbiome testing. The explosion of multi-omics data will enable the development of novel algorithms to predict the impact of the individual’s microbiome on chronic medications, to achieve the best therapeutic outcome [25,96,118,119].

## Figures and Tables

**Table 1:** Susceptible medications for microbial metabolism.

Microbe	Drug	Microbial Metabolite	Metabolic Reaction	Reference
Unknown GI bacterial species	Prontosil Neoprontosil	Sulfanilamide	Azo reduction	[30,31]
Unknown GI bacterial species	Sulfasalazine Balsalazide Olsalazine	5-aminosalicylic acid	Azo reduction	[30,32,33,34]
*Clostridium leptum*	Nitrazepam Clonezepam	7-amino clonazepam	Nitro reduction	[30,39,40]
*Eubacterium lentum*	Digoxin	Dihydrodigoxin	Reduction	[30,40]
Unknown GI bacterial species	Loperamide Oxide	Loperamide	N-oxide bond cleavage	[30,35]
*Eubacterium*	Sodium picosulfate	4,4′-dihydroxy diphenyl-(2-pyridyl)-methane	Hydrolysis	[30,36]
Enterobacteriaceae, primarily	Irinotecan	Glucuronidase enzyme	Hydrolysis	[30,37]
*Bacteroidetes* and *Clostridium* species	Levamisole	Levametabol-I, II, III	Oxidation	[30,31,38]
Unknown GI bacterial species	Insulin Calcitonin	Proteolytic enzymes	Peptide degradation	[30,41]
*Helicobacter pylori*	Levodopa	Cell adhesions	Epithelial cell binding	[30,42]
*Escherichia coli*	Baicalin	Baicalein	Hydrolysis	[30,43,44,45]
*Bifidobacterium bifidum*	Hesperidin	Aglycones hesperetin	Hydrolysis	[30,46]
*Eubacterium rectale* *Streptococcus faecium*	Daidzin	Daidzein	Hydrolysis	[30,47,48]

**Table 2:** The impact of various drugs on the abundance of microbiota.

Microbiota Shift	Drug Type	References
*↑* (increase) *Streptococcus salivarius*, *Lactobacillaceae*, *Eubacteriaceae*	Opiates	[88,91]
*↑ Firmicutes* *Akkermansia muciniphila*, *Faecalibacterium prausnitzii* ↓ (decrease) *Proteobacteria * *Enterobacteriaceae* *Desulfovibrio*, *Prevotella* *Streptococcus*, *Collinsella*	Atorvastatin	[20,91]
*↓ Bacteroidetes*	Methotrexate	[89]
*↑ Actinomyces* *Clostridium leptum*	L-thyroxine	[88,90]
*↑ Streptococcus salivarius* *Rothia*	Oral steroids	[88]
*↑ Streptococcus salivarius*	Platelet aggregation inhibitors	[88,91]
*↑ Streptococcus salivarius*	Vitamin D supplements	[23,88]
*↑ Haemophilus parainfluenzae*	Benzodiazepine	[88]
*↑ Bifidobacterium dentium*, *Streptococcus salivarius*	Proton pump inhibitors	[88,91]
*↑ Eubacterium ramulus*, *Streptococcus salivarius*	SSRI antidepressants	[88,91]
*↑ Clostridium leptum*	Tricyclic antidepressants	[88,91]
*↑ Alistipes* and *Bacteroides*	Laxatives	[88,91]
*↑ Streptococcus mutans* *Bifidobacterium dentium*	Steroid inhalers	[88]
*↑ Escherichia coli*, *Streptococcaceae* *Akkermansia*, *Alistipes* *↓ Lactonifactor*, *Odoribacter*, *Lactococcus*, *Blautia*, *Bacteroides*	Metformin	[84,88,91]

**Table 3:** Database and Prediction Software.

Database/Software Name	Search Result/Prediction	Reference
Database		
MASI: Microbiota Active-Substance Interaction Database	Information on: Transformation of bioactive substances by gut microbes and vice versa. Disease and microbe interactions.	[93]
MagMD: Metabolic action of gut Microbiota to Drugs	Similar information to MASI, but inclusive of the information on enzyme name and effect on drug efficacy.	[94]
Prediction Software		
DrugBug	List of microbes with metabolic enzymes suggested to degrade the drug.	[95]
Machine learning model	Predicts the effect of administered drugs on the microbiome. Predicts if the drug will be depleted/not depleted.	[96,97]
